# Hydroxyapatite-Based Coatings on Silicon Wafers and Printed Zirconia

**DOI:** 10.3390/jfb15010011

**Published:** 2023-12-27

**Authors:** Antoine Chauvin, Marie-Rose Garda, Nathan Snyder, Bai Cui, Nicolas Delpouve, Li Tan

**Affiliations:** 1 Groupe de Physique des Matériaux UMR 6634, CNRS, Université de Rouen Normandie, INSA Rouen Normandie, F-76000 Rouen, Francemarie-rose.garda@univ-rouen.fr (M.-R.G.); 2 Department of Mechanical and Materials Engineering, University of Nebraska, Lincoln, NE 68588, USAbcui@unl.edu (B.C.); ltan4@unl.edu (L.T.)

**Keywords:** HAp, additive manufacturing, thermal properties, microscopy, sol–gel dip coating

## Abstract

Dental surgery needs a biocompatible implant design that can ensure both osseointegration and soft tissue integration. This study aims to investigate the behavior of a hydroxyapatite-based coating, specifically designed to be deposited onto a zirconia substrate that was intentionally made porous through additive manufacturing for the purpose of reducing the cost of material. Layers were made via sol–gel dip coating by immersing the porous substrates into solutions of hydroxyapatite that were mixed with polyethyleneimine to improve the adhesion of hydroxyapatite to the substrate. The microstructure was determined by using X-ray diffraction, which showed the adhesion of hydroxyapatite; and atomic force microscopy was used to highlight the homogeneity of the coating repartition. Thermogravimetric analysis, differential scanning calorimetry, and Fourier transform infrared spectroscopy showed successful, selective removal of the polymer and a preserved hydroxyapatite coating. Finally, scanning electron microscopy pictures of the printed zirconia ceramics, which were obtained through the digital light processing additive manufacturing method, revealed that the mixed coating leads to a thicker, more uniform layer in comparison with a pure hydroxyapatite coating. Therefore, homogeneous coatings can be added to porous zirconia by combining polyethyleneimine with hydroxyapatite. This result has implications for improving global access to dental care.

## 1. Introduction

The last few decades have seen a growing demand for dental surgery as the population increases and ages, and the market for dentistry implants is expected to significantly increase [1]. The most notable current targets of improvement for dental reconstruction are related to implant design and stability [2,3,4,5,6,7,8,9,10], material choice optimization [11,12,13,14,15,16,17], osseointegration [9,10,18,19,20], biocompatibility [13,14,21,22,23], soft tissue integration [24,25,26,27,28,29,30], and healing activity [31,32]. Titanium and zirconia are the most frequently used scaffolds. Zirconia is the more stable candidate mechanically and chemically, and it also exhibits aesthetic advantages (as it has the color of a tooth). Moreover, zirconia-based implants can compete against titanium in terms of bacteria resistance [33,34]. Although zirconia is a favored candidate, its biological inertness limits osseointegration [35,36]. Hydroxyapatite (HAp), the main mineral constituent of dentine and tooth enamel, is commonly used as a coating component, notably for overlaying titanium- or ceramic-based implants. HAp can be successfully added to zirconia to improve dental reconstruction [36]. In brief, HAp has a bone-like mineral composition, and it contributes to osseointegration when it dissolves. The released calcium and phosphorous ions can be used by the surrounding tissues to consolidate the dental implant [37].

Ideal HAp coatings for orthopedic implants are thin (<50 μm), have high cohesive strength to prevent delamination, have a high hardness scale rating to reduce the wear, and are rough with sufficient porosity to promote the ingrowth of bone tissues [38,39,40]. Sol–gel coating [41], pulsed laser deposition [42], pulsed electrochemical deposition [43], pulsed plasma deposition [44], atmospheric plasma spraying [45], and suspension plasma spraying [46] are some examples of deposition techniques. Atmospheric plasma spraying is the most common thermal spray process for fabricating HAp coatings due to its relatively low cost and ability to coat large areas of complex shapes [40]. On the other hand, it was reported that the design of thin coating via atmospheric plasma spraying leads to phase decomposition and poor adhesion strength [38,40]. Moreover, this process could introduce local heating, which can degrade HAp or induce phase transformation [36,47]. 

Sol–gel dip coating [48,49,50] is a low-temperature alternative that exhibits advantages in terms of flexibility and biocompatibility. Some of its merits include a simple setup, flexibility with irregularly shaped substrates, and easy control of both thickness and porosity. Because it requires minimal consumption of materials, it is easy to coat a wide range of substrates with bioactive and biocompatible materials [51]. The substrate is simply dipped into a starting colloidal solution (the ‘sol’) and is then withdrawn at a constant speed to enable solution drainage and instantaneous gelation. The thickness and morphology of coatings can be adjusted by varying the substrate withdrawal speed and viscosity of the solution [50].

This present study considers a zirconia scaffold that is manufactured via additive manufacturing (3D printing). This process can realize complex designs, which enables precise control over porosity for the benefits of osseointegration [40] while reducing the cost of materials. Unfortunately, some complications emerge with the coating of HAp, especially regarding dewetting and the formation of a discontinuous layer. These issues are, for the first time, investigated by blending a sol–gel solution with polyethyleneimine (PEI). PEI is a versatile polymer that exists in a linear and branched form and can act as a ligand with several molecules. It has attracted attention from the paper industry [52], from the electronic domain [53], and in heat exchange membranes [54]. It is used biomedically as a transfection agent for gene delivery [55] and stabilizer for HAP nanoparticles for drug delivery [56]. On the other hand, PEI is not completely harmless to the human body: it is cytotoxic at high concentrations and can cause cell apoptosis [55]. 

Therefore, several tests were attempted to remove the excess PEI. The first tests were performed on HAp-coated wafers of silicon, which are less expensive than zirconia. Following this procedure, thermogravimetric analysis (TGA), differential scanning calorimetry (DSC), and Fourier transform infrared spectroscopy (FTIR) were performed on the annealed materials to record the possible residual signatures of PEI. Then, the HAp coating microstructure and morphology were characterized via X-ray diffraction (XRD) and atomic force microscopy (AFM). Finally, the proper coating of HAp on zirconia porous ceramics was verified using scanning electron microscopy (SEM). To the authors’ knowledge, this is the first time that this strategy has been proposed for the coating of HAp, that is, combining the design of porous zirconia scaffolds, which are made using digital light processing (DLP), with an adapted deposition method based on mineral-organic interactions. Thus, the aim of this study is to develop the PEI-HAp coating to improve the adhesion of hydroxyapatite to zirconia.

## 2. Materials and Methods

### 2.1. Preparation of Zirconia Substrate

The preparation of printed supports was performed via digital light processing (DLP). Its technology is similar to stereolithography, but it uses a UV projector as a light source, which enables it to cure a complete layer of resin at a time. This process significantly increases the rate of manufacturing. The practice of applying this technology to zirconia ceramic printing has been widely presented in the literature, including for applications in the dental industry [57,58,59,60,61]. The protocol followed in this study was reported by Snyder [62]. Oleic acid dispersant and ceramic powder were mixed in ethanol before the solvent was removed. For the DLP method, a slurry, or semi-liquid mixture, must be created to make the ceramic green body (GB). The GB is the ceramic body that results after printing. It is composed of ceramic particles that are held together by a cured polymer, and it must be heat treated further to create a dense ceramic part. 

The build plate descends into the slurry, and a UV light projector shines up from below, projecting the curing layer image. After lifting, the build plate waits for resin to refill, then returns to the vat with the adjusted layer thickness and cures the next layer. The subsequent debinding process, the initial heating stage post-sintering, removes the polymer binder, leaving ceramic particles in the body. Finally, the system undergoes sintering, with the local temperature increased to 1500 °C.

### 2.2. HAp Solution 

To prepare the HAp solution, 0.015 mol (2.494 g) of triethylphosphite (TEP, Thermo Scientific Alfa Aesar, Ward Hill, MA, USA) was added to a mixed solution of 23.92 mL ethanol and 1.08 mL deionized water. Then, 0.0251 mol (4.1102 g) of calcium nitrate tetrahydrate (Ca(NO_3_)_2_·4H_2_O, Thermo Scientific Alfa Aesar, USA) was dissolved in 25 mL ethanol. These solutions were mixed at room temperature for 3 days and aged at 37 °C for 1 day with continuous stirring to make the HAp sol. The theoretical concentration of the final HAp sol was 0.05 M when it was assumed that all reactants completely reacted. 

### 2.3. PEI-HAp Solution 

First, 0.625 × 10^−3^ mol (500 mg) of branched PEI (Sigma Aldrich 408727, Saint Louis, MO, USA, CAS number = 9002–98–6), was added to a solution of 50 mL of deionized water. This solution was stirred at 80 °C for one minute. Then, this solution was mixed with HAp solution under a ratio of 1:1 in a 10 mL plastic test tube.

### 2.4. PEI-HAp Coating on Zirconia

The PEI-HAp was coated with a laboratory dip-coating equipment. The dip-coating rate was 0.25 mm/s and the experiments were performed thrice. The sol was dried for 5 min in the oven at 37 °C between each dipping step. The annealing was performed after the coating was completed. The PEI-HAp-coated zirconia sample was oven-dried at 70 °C overnight and annealed at 230 °C for 1 h (to degrade the PEI) then at 800 °C for 1 h. For both ramps, the heating rate was 5 °C/min. Before any characterization, the coating adhesion was tested by sticking a piece of tape on the substrate, then removing it from several angles to mimic tearing solicitations. Only samples without coating damage were kept for investigation. An example is provided in Supplementary Materials (Figure S1). 

### 2.5. Characterization of the PEI-HAp Coating

A Dimension 3100 SPM System along with a Digital Instrument Nanoscope IIIa was used for the atomic force microscopy (AFM). This system was set to contact mode, applying a constant force of 0.1 N/m. The measurement was performed with a resolution of 0.5 nm and an accuracy of 1 nm. 

Thermogravimetric analyses (TGAs) were carried out using a TGA Discovery instrument from TA Instruments^®^, Guyancourt, France. The analyses were carried out under nitrogen atmosphere at a 25 mL min^−1^ flow rate, in the temperature range of 30–800 °C and a scanning rate of 10 K min^−1^ on 2 mg samples. The apparatus calibration procedure included a baseline, mass, and temperature calibration. The temperature calibration was performed using the Curie point of nickel as reference. The mass calibration was performed using standard masses, and the calibration in mass loss was performed using calcium oxalate as the reference.

The differential scanning calorimetry (DSC) experiments were conducted on a DSC Q100 from TA Instruments^®^, Guyancourt, France. The experiments were conducted on samples of 2 mg mass at 10 K/min, and under nitrogen atmosphere at a 50 mL min^−1^ flow rate. The calibration procedure includes a baseline with an empty furnace. To calibrate the temperature and energy, a standard sample of indium (T_m_ = 156.60 °C and ΔH_m_ = 28.38 J g^−1^ corresponding to melting temperature and enthalpy respectively) was used. 

The infrared spectroscopic details were recorded on a Thermo Fischer^®^ (Montigny-le-Bretonneux, France) Nicolet iS10 in transmission mode in the 425–4000 cm^−1^ range, with a resolution of 4 cm^−1^. A background correction was performed prior to the experiment. The analyses of IR spectra were performed using the apparatus Omnic 9.3. software.

The X-ray diffraction (XRD) analyses were performed using a Rigaku^®^ Multiflex Diffractometer (Austin, TX, USA). The Rigaku Multiflex consists of a 2 kW copper tube and a θ/θ goniometer. The sample holder remains horizontal during the scan and therefore, there is no need to use adhesive substances to mount samples on to a low background sample holder plate. It is also configured in focusing geometry where a secondary monochromator removes the scattered signal except that corresponding to Cu Kalpha wavelength. The samples analyzed via XRD were under the form of powders that were obtained by heating the HAp solutions at 80 °C to boil ethanol. 

The scanning electron microscopy (SEM) pictures were acquired using a Hitachi^®^ (Schaumburg, IL, USA) S4700 field-emission SEM with a high-resolution digital processing system. The work distance was in the order of 20 nm. The sample was fractured in liquid nitrogen, then polished and fixed on the support with a conductive coating.

## 3. Results and Discussion

### 3.1. Coating Topography

**Figure 1:**
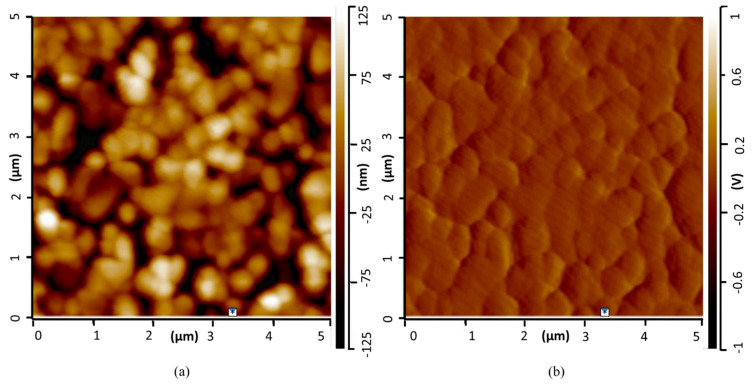
AFM picture of the silica wafer surface that was coated using HAp-PEI, then annealed: (**a**) topography relief of the coating; (**b**) deflection signal related to the AFM measurement.

The surface relief is an important element to validate the dip-coating experimental conditions. A first glance at the impact of sol–gel deposition on the coating morphology is provided on Figure 1. It shows images produced by AFM measurements of the coating, which was made by mixing HAp with PEI after being overlayed onto the silica wafer and annealed. The AFM probe provides a precise nanoscale measurement of the different cliffs and clusters. Figure 1a provides the topography (the relief depth is ±125 nm on the AFM picture) and Figure 1b provides the deflection signal. A bright point in each image indicates a high value while a dark point indicates a low value. One can observe from the topography images that slight agglomerations of brighter light are dispersed throughout the sample. They are attributed to the coating domain with a higher thickness, while the black parties should indicate the absence of coating in the probed area. One can consider, despite slight irregularities, that the coating is fairly homogeneous. Moreover, one can observe that the surface does not exhibit any cracks.

### 3.2. Coating Characterization

**Figure 2:**
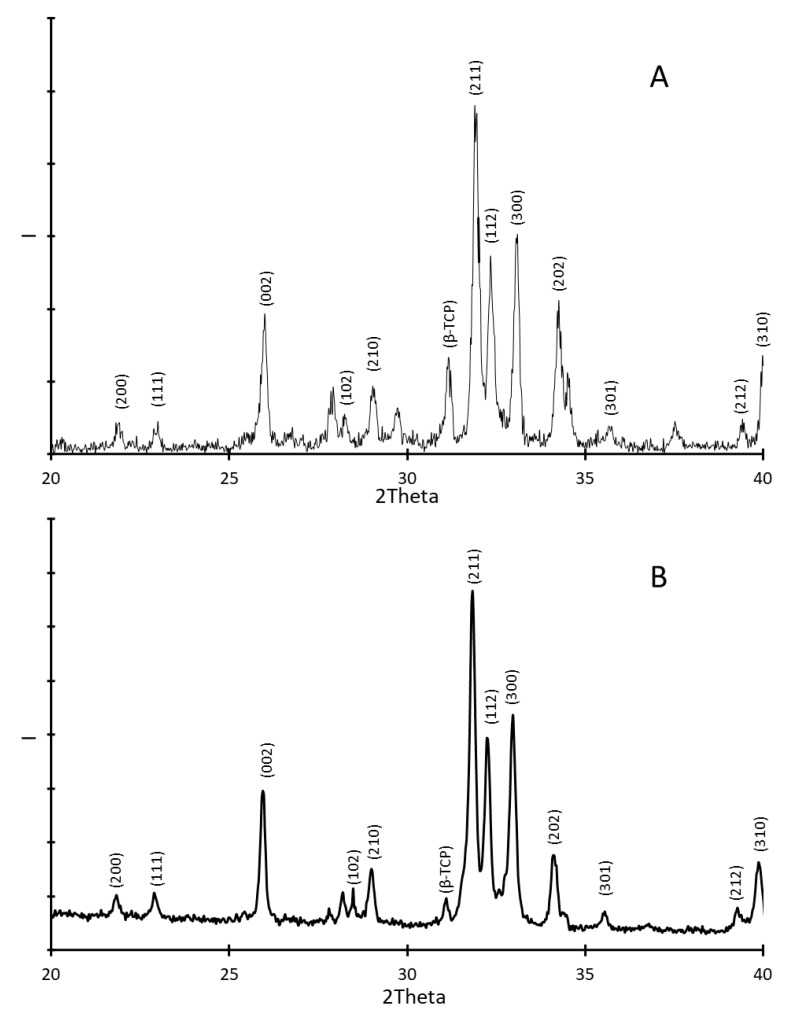
XRD analyses on (**A**) synthesized hydroxyapatite; (**B**) the coating made from the mix of HAp and PEI and deposited on the silica wafer.

To ensure that the annealing procedure did not damage or alter the microstructure of the HAp coating, X-ray analyses were performed. The results were compared with those obtained for pure HAp. These results are shown in Figure 2A for HAp alone and in Figure 2B for the coating on the silica wafer. Thus, according to the spectra, the signature of the coating is very similar to the one of the non-treated HAp and is in agreement with the literature [63]. The peaks of the diffracting planes (200) and (111) are found between 2Theta = 2θ = 20° and 2θ = 25°, with 2θ the angle of deviation between the incoming beam and the reflected beam. The peaks of the highest intensity I are observed between 2θ = 25° and 2θ = 35°. They characterize the diffracting planes (002), (210), (211), (112), (300), and (202). Finally, the peaks corresponding to the diffracting planes (301), (212), and (310) are recorded between 2θ = 35° and 2θ = 40°. This reflects that the annealing did not degrade the HAp coating.

To check whether its composition and crystalline form was preserved after the annealing, it was necessary to track for possible singularities between the two spectra. The most significant difference that can be observed lies in the peak situated around 2θ = 31°, which is characteristic of the presence of β-tricalcium phosphate (β-TCP) [64], one of the most used and potent synthetic bone graft substitutes [65]. This corresponds to the rhombohedral crystalline form of tricalcium phosphate (TCP) [66]. This is the densest TCP polymorph but it is not a stable crystalline form at high temperatures, contrary to both monoclinic α-TCP and hexagonal α’-TCP. The β-TCP peak is clearly visible in the non-treated HAp and barely detectable in the coating. This suggests that the annealing performed up to 800 °C partially or totally decomposes β-TCP. It is worth mentioning that there is no evidence of the presence of α-TCP either. Since the removal or the transformation of β-TCP is expected to impact the residual properties of the implant [67], different annealing procedures should be evaluated to determine which one leads to the most interesting macroscopic behavior regarding the targeted dental applications.

**Figure 3:**
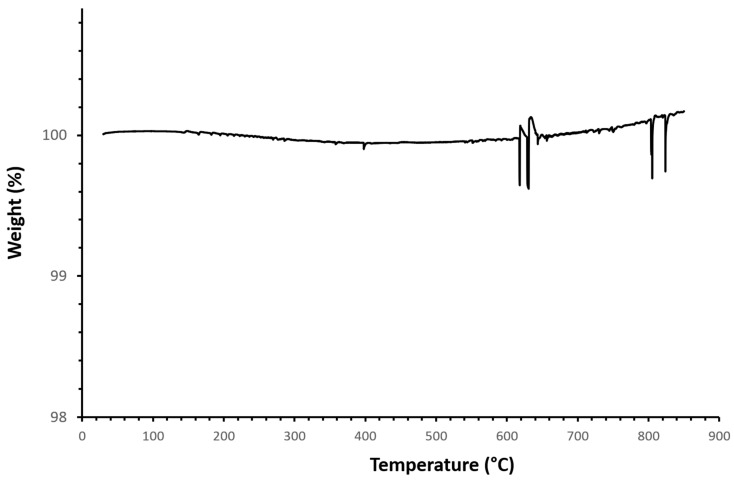
Evolution of the sample mass percentage as a function of temperature from thermogravimetric analyses performed on the silica wafers that were coated by the mix of HAp and PEI, then annealed.

While previous results attest to the coating survival consecutive to the annealing procedure, the removal of PEI, which is expected to degrade at about 300 °C, remains to be verified. According to the TGA results, presented in Figure 3, the process of heating silicon wafers coated with the HAp and PEI mix under a nitrogen atmosphere does not reveal significant loss of mass from the ambient temperature up to 900 °C. Only above 600 °C could some artefacts of measurements be observed, which are probably linked to the impact of high temperature on the sensors. The absence of any degradation signature might indicate that PEI was properly decomposed during the previous annealing step. Nevertheless, it is worth mentioning that data collected from the literature do not necessarily agree concerning the behaviour of HAp when submitted to a temperature ramp.

According to Phuong et al. [68], the total weight loss of HAp when subjected to harsh conditions, that is, heated under air up to 800 °C, is insignificant in comparison to what has previously been reported by other authors [69,70,71], as the results of this study show HAp residue values that were close to 99.5% weight. This slight weight loss was essentially attributed to the evaporation of residual water. On the other hand, the studies performed by the aforementioned authors [69,70,71] reveal more significant traces of degradation of the HAp structure. Lazić et al. [69] identified three stages of HAp degradation. Up to 200 °C, the weight loss is imputed to water evaporation. From 200 to 650 °C, the decomposition of hydrogen phosphate occurs, which can be combined with the loss of interstitial water. Above 650 °C, reactions involving the decomposition of diphosphates take place. The final loss of weight is estimated to be about 7%, but it is already higher than 3% at the end of the first stage, which does not correspond to the results presented there, since it suggests that a more significant amount of water is present in the material before the TGA run. Jagadale et al. [70] reported an almost full HAp degradation into nano-porous powders with a very prominent first stage, but they did not interpret the high-temperature behaviour. Safarzadeh et al. [71] separated the degradation of carbonated Hap into two stages. The first stage is essentially caused by the liberation of absorbed and lattice water, which accounts for weight loss up to 12%. The second stage is less pronounced and was assumed to be linked to the decomposition of carbonates. 

These strong inconsistencies between reports in terms of interpretations and results lead us to two possible assumptions regarding our results. First, it assumed that the sample weight remains constant throughout the analysis, as could be expected from any scan performed on HAp-coated silicon. In this case, annealing at 800 °C should have completely removed PEI from the substrate. In addition, the absence of water loss below 200 °C can be easily explained by the absence of residual water into the material, due to the annealing step performed prior to analysis. Nevertheless, this is not the only case that could be considered. The apparatus may not have detected degradation due to resolution issues. In this latter case, the absence of weight loss would not prove that PEI effectively disappeared during the annealing step that was performed before TGA. Although this interpretation seems less likely, other characterisations were performed to consider additional elements.

**Figure 4:**
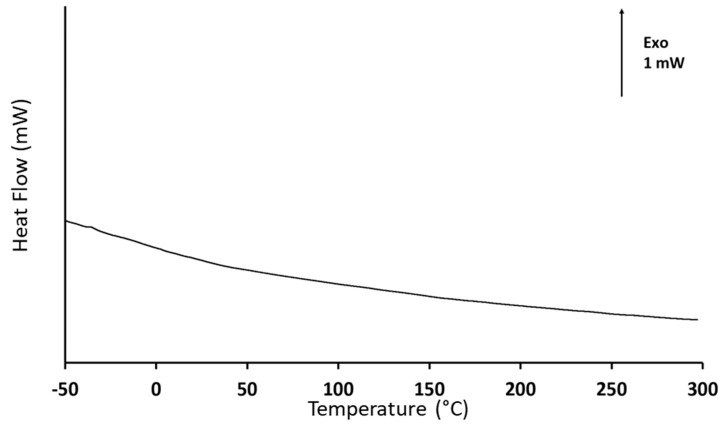
Differential scanning calorimetry analyses performed on the silica wafers that were coated by the mix of HAp and PEI, then annealed: evolution of the heat flow between sample and empty furnace as a function of temperature.

The DSC results, shown in Figure 4, lead to similar interrogations. According to the heat flow variations with temperature, there is no evidence of any transition in the temperature range covered by the analysis. The events of interest for this analysis are the PEI glass transition and its onset of decomposition. Typically, the glass transition of PEI is expected to be around −25 °C [72], while the thermal decomposition has been reported to start at about 250 °C [73]. However, the obtained DSC response is identical to the signal obtained when performing the baseline step during the calibration procedure. This observation is consistent with the results reported by Feng et al. [74] for a plasma-sprayed Ti-6Al-4V/HAp composite. In this study, no calorimetric event was recorded before 700 °C, that is, before the phase transformation of HAp. The absence of any characteristic thermal event is also consistent with TGA results. Thus, it is reasonable to assume that there is no residual PEI on the sol–gel dip coated support, due to the annealing procedure. Nevertheless, it is worth reminding that the quantity of PEI mixed with HAp is very small, as its role lies essentially in facilitating the adhesion of HAp on the support. Thus, the detection of residual traces can be particularly challenging and possibly beyond the resolution limits of both thermal analysis apparatuses.

**Figure 5:**
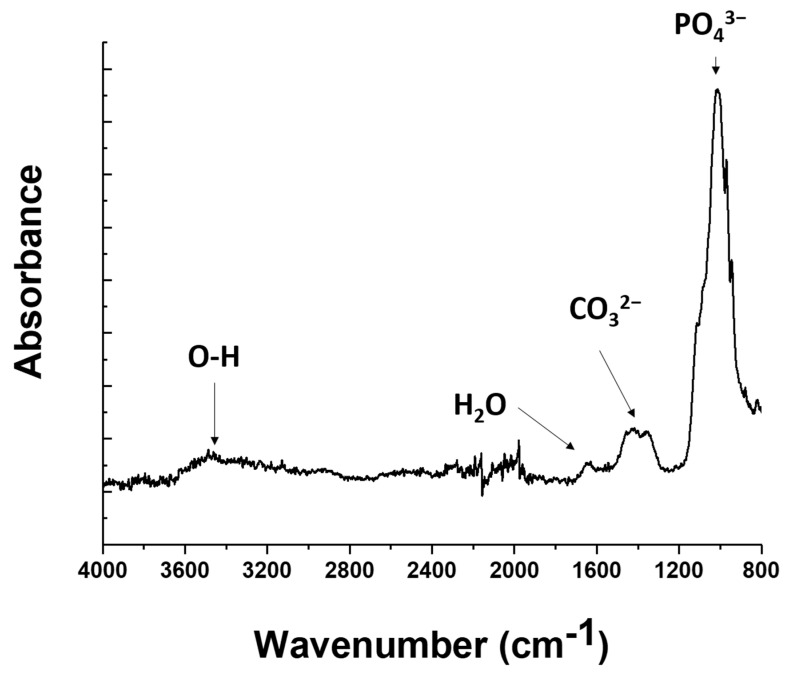
Infrared spectrum analysis on the silica wafers that were coated by the mix of HAp and PEI.

Therefore, the results obtained from thermogravimetric and calorimetric investigations were complemented with infrared spectroscopy analyses. The resulting spectrum is presented in Figure 5. The PEI infrared signature [75] is classically characterized by the N–H stretching band at 3300 cm^−1^, the C–H stretching bands around 3000 cm^−1^, the C–H stretching bands around 3000 cm^−1^, the bending vibration of the CH2 group at 1500 cm^−1^, and the C–N stretching band around 1100 cm^−1^. No peak was recorded at these characteristic wavenumbers. On the other hand, the obtained spectrum is in good agreement with references from the literature describing the infrared signature of hydroxyapatite [76,77], which confirms the adhesion of the coating, as shown in Figure 2. At about 1050 cm^−1^, a main intense peak is recorded, which can be attributed to the asymmetric stretching vibration of phosphate P–O bonds. Around 1400 cm^−1^, one can find the broad signature of C–O bond asymmetric stretching from carbonate ions. The small peak above 1600 cm^−1^ belongs to residual water according to Prekajski et al. [78]. Finally, the broad peak located between 3200 and 3600 cm^−1^ is linked to O–H bonds, but its intensity remains weak, probably due to the strong annealing procedure performed prior to the material characterization. The unambiguous infrared signature of hydroxyapatite indicates that the infrared spectroscopy is adequate to investigate the composition of the coating. Due the absence of band characteristics of PEI, one can think that the annealing treatment successfully removed any trace of PEI. 

### 3.3. Coating Morphology on Printed Zirconia

**Figure 6:**
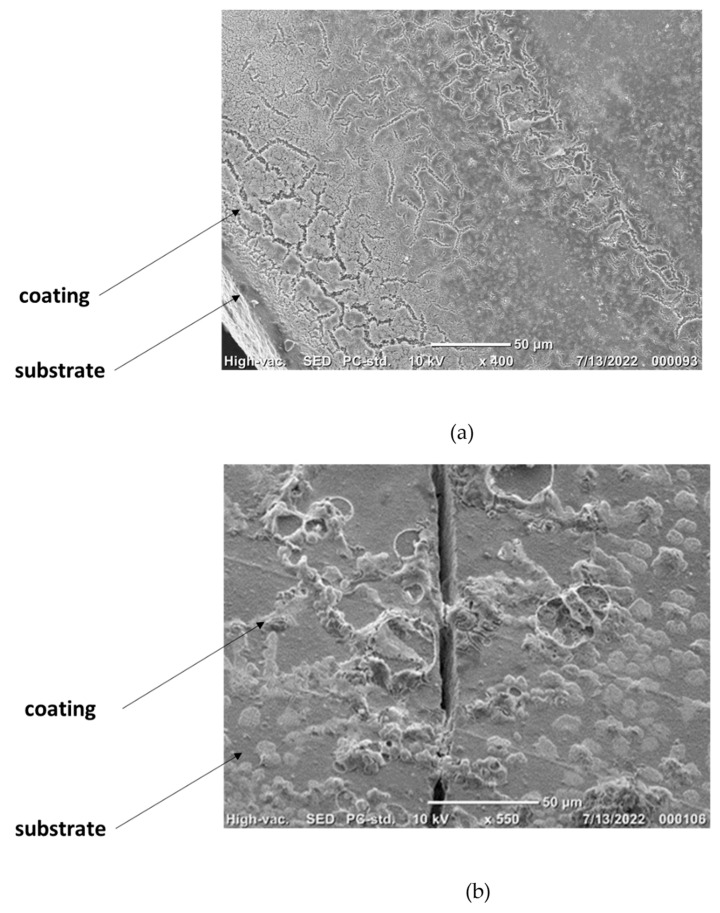
SEM pictures of coated porous zirconia ceramic obtained by additive manufacturing: (**a**) cross-sectional view of HAp/PEI coating on zirconia annealed at 800 °C; (**b**) bare HAp on printed zirconia.

The HAp coating was seemingly preserved after the removal of PEI. A conceptual test was performed by comparing coatings of HAp alone and HAp mixed with PEI on 3D-printed zirconia. Zirconia is a commonly used material in dentistry implants, and its compatibility with this HAp coating is a main requirement for future applications. SEM pictures of both systems are presented in Figure 6. These describe the coating morphology and adhesion for each case. The picture presented in Figure 6a was obtained after analysing 3D-printed zirconia coated with the HAp and PEI mixture. It shows the cross-section between a coated and an uncoated part after the sample was cut in two. The coating is clearly visible, and it apparently takes the form of the porous structure obtained by the rapid prototyping procedure. It is not perfectly uniform, in comparison with reports from literature regarding dense systems, for example the report by Jiang et al. [79]. However, a clear improvement is noticeable when compared with the HAp coating alone (Figure 6b), which exhibits a much-disorganised repartition of the coating with insulated portions of HAp facing many wide empty spaces. Moreover, the bare-coated sample also exhibits a craze that may indicate a more fragile structure in the absence of ligand polymer. Therefore, a clear improvement of the coating adhesion is assessed when HAp is coupled with PEI before being deposited. In other terms, HAp coating alone is not sustainable on 3D-printed zirconia; thus, HAp-PEI is proposed as a coating instead. It is worth mentioning that the HAp-PEI does not fully cover the surface of the substrate either. This could be related to the quantity of material being too low. It is expected that a higher grafted quantity of material would lead to a more continuous coating. 

### 3.4. Benefits and Drawbacks of DLP and Organic–Mineral Interactions for HAp Coating

The long-term objective behind this work is to facilitate the access to dental care by proposing a protocol that is transferable to the industrial scale that compensates the technological cost related to the scaffold design by the reduction in global content of matter. Therefore, it is important to critically evaluate the properties of HAP coatings on DLP porous substrates. The process also includes the mixing of HAp with PEI to improve the homogeneity and the sustainability of the coating. This step, based on the interactions between mineral and organic compounds, has been shown to be of value as it significantly improves the continuity of the coating. In Table 1, the main characteristics of this HAp coating are summarized and compared with others from the literature, either on plain or porous scaffolding. 

## 4. Conclusions

In this study, a dip-coating procedure effectively deposited PEI-HAp onto the surface of 3D-printed zirconia. The coating of HAp on porous substrates is not uniform and its adhesion is poor. The results are significantly better when HAp is mixed with PEI. The adhesion of HAp mixed with PEI is possible on several surfaces including silica and zirconia in the present case. A subsequent annealing procedure successfully removes PEI. It does not damage the HAp coating.

The interest of this procedure lies in the relative conservation under high-temperature annealing of the HAp structural integrity while destructing PEI. Consecutively to the annealing, the hydroxyapatite signature is indeed clearly visible via X-ray diffraction and infrared spectroscopy whereas the signature of the polyethyleneimine is not recorded by TGA, DSC, or IR. Therefore, not only does PEI improve the adhesion of HAp, but it can also be successfully removed without significantly damaging the HAp coating. 

Several immediate conclusions result from this work:

- Zirconia substrate obtained via digital light processing (DLP) can be used as a scaffold for coating deposition.
- The coating of HAp on porous substrates is not uniform and its adhesion is poor. The results are significantly better when Hap is mixed with PEI.
- The adhesion of HAp mixed with PEI is possible on several surfaces including silicon and zirconia in the present case.
- A subsequent annealing procedure successfully removes PEI. It does not damage the HAp coating.

It is worth mentioning that the possibility of interactions between HAp and PEI remains to be investigated. Future works should also consider further examination of the removal of polyethyleneimine, adhesion tests, and toxicity controls.

## Figures and Tables

**Table 1:** Features of the designed coating procedure in comparison with other methodologies from previous studies.

Methodology	Objectives	Results
Pulsed laser deposition [42]	- Controlled thickness - Controlled transfer at a stoichiometric level of the composition of the material - Good adherence	- Coating morphology tailorable by varying the incident energy - Microhardness values of the hydroxyapatite coating increase with the incident energy - Good stoichiometry
Pulsed electrochemical deposition [43]	- Improved compositional control - Coating uniformity - Versatility - Low-cost procedure	- High control in the amount, purity, and homogeneity of the coating - Morphology influenced by current density
Pulsed plasma deposition [44]	- Improved tribological properties - Wear resistance	- Design of coated spheres - Sub-micrometric grain size distribution - Low roughness - Lower wear rate in comparison with uncoated systems
Atmospheric plasma spraying [45]	- Design of bioactive glass - Improved bond strength by combining bioactive glass with HAp	- Improvement of adhesion strength by thermal treatment - Coating properties influenced by the amorphous/crystal ratio
Suspension plasma spraying [46]	- Improved mechanical properties	- Uniform microstructure - Low porosity - Enhanced mechanical properties
Axial suspension plasma spraying [40]	- Improved adhesion strength - Limited phase degradation	- Successful design of coatings with high adhesion strength - The lower solid load content and lower mean solute particle size in the suspension was found to be beneficial to achieve porous and rougher coatings
In-situ sol–gel process [41]	- Improved corrosion behaviour - Improved in vitro activity	- Homogenous deposition - Compact and crack-free coating - Surface roughness, adhesion strength and hydrophilicity are tailorable - Good performances in terms of corrosion and bioactivity
DLP + dip-coating [this study]	- Rapid substrate design - Design of a porous scaffold to facilitate the osseointegration - Reducing of the material cost	- Successful design of porous zirconia scaffold - HAp alone does not adhere on the substrate - Adhesion improved by PEI - Sustainability of the coating post-annealing

## Data Availability

Data are available in this article.

## Supplementary Materials

The following supporting information can be downloaded at: https://www.mdpi.com/article/10.3390/jfb15010011/s1, Figure S1, Surface state consecutive to the removal of a piece of tape stuck on the coating.

## References

[1] Londoño J.J., Ramos A.M., Correa S.A., Mesnard M. (2021). Review of expandable dental implants. Brit. J. Oral Max. Surg..

[2] De Stefano M., Lanza A., Faia E., Ruggiero A. (2023). A novel ultrashort dental implant design for the reduction of the bone stress/strain: A comparative numerical investigation. Biomed. Eng. Adv..

[3] Panchal M., Khare S., Khamkar P., Bhole K.S. (2022). Dental implants: A review of types, design analysis, materials, additive manufacturing methods, and future scope. Mater. Today Proc..

[4] Matsko A., França R. (2022). Design, manufacturing, and clinical outcomes for additively manufactured titanium dental implants: A systematic review. Dent. Rev..

[5] Yin S., Zhang W., Tang Y., Yang G., Wu X., Lin S., Liu X., Cao H., Jiang X. (2021). Preservation of alveolar ridge height through mechanical memory: A novel dental implant design. Bioact. Mater..

[6] Moreira A., Madeira S., Buciumeanu M., Fialho J., Carvalho A., Silva F., Monteiro F.J., Caramês J. (2022). Design and surface characterization of micropatterned silica coatings for zirconia dental implants. J. Mech. Behav. Biomed..

[7] Dantas T.A., Pinto P., Vaz P.C.S., Silva F.S. (2020). Design and optimization of zirconia functional surfaces for dental implants applications. Ceram. Int..

[8] Zanetti E.M., Pascoletti G., Cali M., Bignardi G., Franceschini G. (2018). Clinical Assessment of Dental Implant Stability During Follow-Up: What Is Actually Measured, and Perspectives. Biosensors.

[9] Kittur N., Oak R., Dekate D., Jadhav S., Dhatrak P. (2021). Dental implant stability and its measurements to improve osseointegration at the bone-implant interface: A review. Mater. Today Proc..

[10] Qin J., Yang D., Maher S., Lima-Marques L., Zhou Y., Chen Y., Atkins G.J., Losic D. (2018). Micro- and nano-structured 3D printed titanium implants with a hydroxyapatite coating for improved osseointegration. J. Mater. Chem. B.

[11] Olander J., Ruud A., Wennerberg A., Stenport V.F. (2022). Wear particle release at the interface of dental implant components: Effects of different material combinations. An in vitro study. Dent. Mater..

[12] Das I., Chattopadhyay S., Mahato A., Kundu B., De G. (2016). Fabrication of a cubic zirconia nanocoating on a titanium dental implant with excellent adhesion, hardness and biocompatibility. RSC Adv..

[13] Bose S., Koski C., Vu A.A. (2020). Additive manufacturing of natural biopolymers and composites for bone tissue engineering. Mater. Horiz..

[14] Zou R., Bi L., Huang Y., Wang Y., Wang Y., Li L., Liu J., Feng L., Jiang X., Deng B. (2023). A biocompatible silicon nitride dental implant material prepared by digital light processing technology. J. Mech. Behav. Biomed..

[15] Hasan J., Bright R., Hayles A., Palms D., Zilm P., Barker D., Vasilev K. (2022). Preventing peri-implantitis: The quest for a next generation of titanium dental implants. ACS Biomater. Sci. Eng..

[16] Gallegos S.I., Parsaei S., Siddiqui D.A., Biguetti C.C., Palmer K.L., Rodrigues D.C. (2019). Can dental cement composition affect dental implant success?. ACS Biomater. Sci. Eng..

[17] Milone D., Fiorillo L., Alberti F., Cervino G., Filardi V., Pistone A., Cicciù M., Risitano G. (2022). Stress distribution and failure analysis comparison between Zirconia and Titanium dental implants. Procedia Struct. Integr..

[18] Kitagawa I.L., Miyazaki C.M., Pitol-Palin L., Okamoto R., de Vasconcellos L.M.R., Constantiono C.J.L., Lisboa-Filho P.N. (2021). Titanium-based alloy surface modification with TiO2 and poly(sodium 4-styrenesulfonate) multilayers for dental implants. ACS Appl. Bio Mater..

[19] Kim W.-H., Shin Y.C., Lee S.-H., Lee S.-H., Kang M.S., Lee M.-S., Lee J.H., Lee J.-H., Han D.-W., Kim B. (2022). Dental implants with electrochemical nanopattern formation to increase osseointegration. J. Ind. Eng. Chem..

[20] Wu Y., Feng F., Xin H., Li K., Tang Z., Guo Y., Qin D., An B., Diao X., Dou C. (2019). Fracture strength and osseointegration of an ultrafine-grained titanium mini dental implant after macromorphology optimization. ACS Biomater. Sci. Eng..

[21] Gautam C., Joyner J., Gautam A., Rao J., Vajtai R. (2016). Zirconia based dental ceramics: Structure, mechanical properties, biocompatibility and applications. Dalton Trans..

[22] Ma M., Zhao M., Deng H., Liu Z., Wang L., Ge L. (2023). Facile and versatile strategy for fabrication of highly bacteriostatic and biocompatible SLA-Ti surfaces with the regulation of Mg/Cu coimplantation ratio for dental implant applications. Colloids Surf. B.

[23] Yilmaz E., Çalişkan F. (2022). A new functional graded dental implant design with biocompatible and antibacterial properties. Mater. Chem. Phys..

[24] Jayasree A., Gómez-Cerezo M.N., Verron E., Ivanovski S., Gulati K. (2022). Gallium-doped dual micro-nano titanium dental implants towards soft-tissue integration and bactericidal functions. Mater. Today Adv..

[25] Ren X., van der Mei H.C., Ren Y., Busscher H.J. (2019). Keratinocytes protect soft-tissue integration of dental implant materials against bacterial challenges in a 3D-tissue infection model. Acta Biomater..

[26] Mühl A., Szabó P., Krafcsik O., Aigner Z., Kopniczky J., Nagy A., Marada G., Turzó K. (2022). Comparison of surface aspects of turned and anodized titanium dental implant, or abutment material for an optimal soft tissue integration. Heliyon.

[27] Kim J.C., Lee M., Yeo I.-S.L. (2022). Three interfaces of the dental implant system and their clinical effects on hard and soft tissues. Mater. Horiz..

[28] Yang Z., Liu M., Yang Y., Zheng M., Yang Y., Liu X., Tan J. (2020). Biofunctionalization of zirconia with cell-adhesion peptides via polydopamine crosslinking for soft tissue engineering: Effects on the biological behaviors of human gingival fibroblasts and oral bacteria. RSC Adv..

[29] Matter M.T., Maliqi L., Keevend K., Guimond S., Ng J., Armagan E., Rottmar M., Herrmann I.K. (2021). One-step synthesis of versatile antimicrobial nano-architected implant coatings for hard and soft tissue healing. ACS Appl. Mater. Interfaces.

[30] Fischer N.G., Moussa D.G., Skoe E.P., De Jong D.A., Aparicio C. (2020). Keratinocyte-specific peptide-based surfaces for hemidesmosome upregulation and prevention of bacterial colonization. ACS Biomater. Sci. Eng..

[31] Fischer N.G., Munchow E.A., Tamerler C., Bottino M.C., Aparicio C. (2020). Harnessing biomolecules for bioinspired dental biomaterials. J. Mater. Chem. B.

[32] De Avila E.D., Castro A.G.B., Tagit O., Krom B.P., Lőwik D., van Well A.A., Bannenberg L.J., Vergani C.E., van den Beucken J.J.J.P. (2019). Anti-bacterial efficacy via drug-delivery system from layer-by-layer coating for percutaneous dental implant components. Appl. Surf. Sci..

[33] Scarano A., Piattelli M., Caputi S., Favero G.A., Piattelli A. (2004). Bacterial adhesion on commercially pure titanium and zirconium oxide disks: An in vivo human study. J. Periodontol..

[34] do Nascimento C., Pita M.S., Fernandes F.H.N.C., Pedrazzi V., de Albuquerque Junior R.F., Ribeiro R.F. (2014). Bacterial adhesion on the titanium and zirconia abutment surfaces. Clin. Oral Implant. Res..

[35] Caravaca C., Shi L., Balvay S., Rivory P., Laurenceau E., Chevolot Y., Hartmann D., Gremillard L., Chevalier J. (2016). Direct silanization of zirconia for increased biointegration. Acta Biomater..

[36] Seesala V.S., Rajasekaran R., Ojha A.K., Mahato A., Korrayi R.R., Das B., Venugopal S.P., Roy S., Dhara S. (2023). A novel functional gradient hydroxyapatite coating for zirconia-based implants. Surf. Coat. Technol..

[37] Gledhill H.C., Turner I.G., Doyle C. (2001). In vitro dissolution behaviour of two morphologically different thermally sprayed hydroxyapatite coatings. Biomaterials.

[38] Heimann R.B. (2016). Plasma-sprayed hydroxylapatite-based coatings: Chemical, mechanical, microstructural, and biomedical properties. J. Therm. Spray Technol..

[39] Bøe B.G., Röhrl S.M., Heier T., Snorrason F., Nordsletten L. (2011). A prospective randomized study comparing electrochemically deposited hydroxyapatite and plasma-sprayed hydroxyapatite on titanium stems. Acta Orthop..

[40] Ganvir A., Nagar S., Markocsan N., Balani K. (2021). Deposition of hydroxyapatite coatings by axial plasma spraying: Influence of feedstock characteristics on coating microstructure, phase content and mechanical properties. J. Eur. Ceram..

[41] Ansari Z., Kalantar M., Kharaziha M., Ambrosio L., Raucci M.G. (2020). Polycaprolactone/fluoride substituted-hydroxyapatite (PCL/FHA) nanocomposite coatings prepared by in-situ sol-gel process for dental implant applications. Prog. Org. Coat..

[42] González-Estrada O.A., Pertuz Comas A.D., Ospina R. (2022). Characterization of hydroxyapatite coatings produced by pulsed-laser deposition on additive manufacturing Ti6Al4V ELI. Thin Solid Films.

[43] Lissandrello F., Magagnin L. (2023). Pulsed electrochemical deposition of calcium phosphate coatings for biomedical applications. Trans. Inst. Mater. Finish..

[44] Bianchi M., Lopomo N., Boi M., Gambardella A., Marchiori G., Berni M., Pavan P., Marcacci M., Russo A. (2015). Ceramic thin films realized by means of pulsed plasma deposition technique: Applications for orthopedics. J. Mech. Med. Biol..

[45] Garrido B., Martin-Morata A., Dosta S., Cano I.G. (2023). Improving the bond strength of bioactive glass coatings obtained by atmospheric plasma spraying. Surf. Coat. Technol..

[46] Chen X., Zhang B., Gong Y., Zhou P., Li H. (2018). Mechanical properties of nanodiamond-reinforced hydroxyapatite composite coatings deposited by suspension plasma spraying. Appl. Surf. Sci..

[47] Oyane A., Kakehata M., Sakamaki I., Pyatenko A., Yashiro H., Ito A., Torizuka K. (2016). Biomimetic apatite coating on yttria-stabilized tetragonal zirconia utilizing femtosecond laser surface processing. Surf. Coat. Technol..

[48] Singh P.P., Dixit K., Sinha N. (2022). A sol-gel based bioactive glass coating on laser textured 316L stainless steel substrate for enhanced biocompatibility and anti-corrosion properties. Ceram. Int..

[49] García-Arnáez I., Cerqueira A., Romero-Gavilán F., Elortza F., Azkargorta M., Iloro I., Suay J., Goñi I., Gurruchaga M. (2022). Development and characterisation of strontium-doped sol-gel coatings to optimise the initial bone regeneration processes. Mater. Today Commun..

[50] Catauro M., Bollino F., Giovanardi R., Veronesi P. (2017). Modification of Ti6Al4V implant surfaces by biocompatible TiO_2_/PCL hybrid layers prepared via sol-gel dip coating: Structural characterization, mechanical and corrosion behavior. Mater. Sci. Eng. C.

[51] Alavi S.E., Panah N., Page F., Gholami M., Dastfal A., Sharma L.A., Shahmabadi H.E. (2022). Hydrogel-based therapeutic coatings for dental implants. Eur. Polym. J..

[52] Vieira M., Tavares C.R., Bergamasco R., Petrus J.C.C. (2001). Application of ultrafiltration-complexation process for metal removal from pulp and paper industry wastewater. J. Membr. Sci..

[53] Lin X., Tran D.T., Song M.-H., Yun Y.-S. (2021). Development of polyethyleneimine-starch fibers stable over the broad pH range for selective adsorption of gold from actual leachate solutions of waste electrical and electronic equipment. J. Clean. Prod..

[54] Li S., Tu L., Lu Y., Lin M., Ma J., Bai C., Gao C., Xue L. (2023). Polyethyleneimine (PEI) based thin film nanocomposite (TFN) total heat exchange membranes (THEMs) composed of shaped ZIF-8 crystalline micro-leaves (ZIF-L). Sep. Purif. Technol..

[55] Zhang C., Wu F.-G., Hu P., Chen Z. (2014). Interaction of polyethylenimine with model cell membranes studied by linear and nonlinear spectroscopic techniques. J. Phys. Chem. C.

[56] Kong L., Mu Z., Yu Y., Zhang L., Hu J. (2016). Polyethyleneimine-stabilized hydroxyapatite nanoparticles modified with hyaluronic acid for targeted drug delivery. RSC Adv..

[57] Li X., Zhong H., Zhang J., Duan Y., Bai H., Li J., Jiang D. (2020). Dispersion and properties of zirconia suspensions for stereolithography. Int. J. Appl. Ceram. Technol..

[58] Borlaf M., Serra-Capdevila A., Colominas C., Graule T. (2019). Development of UV-curable ZrO_2_ slurries for additive manufacturing (LCM-DLP) technology. J. Eur. Ceram. Soc..

[59] Jang K.J., Kang J.H., Fisher J.G., Park S.W. (2019). Effect of the volume fraction of zirconia suspensions on the microstructure and physical properties of products produced by additive manufacturing. Dent. Mater..

[60] Coppola B., Schmitt J., Lacondemine T., Tardivat C., Montanaro L., Palmero P. (2022). Digital light processing stereolithography of zirconia ceramics: Slurry elaboration and orientation-reliant mechanical properties. J. Eur. Ceram. Soc..

[61] Ji S.H., Da S.K., Park M.S., Ji S.Y. (2021). Sintering process optimization for 3YSZ ceramic 3D-printed objects manufactured by stereolithography. Nanomaterials.

[62] Snyder N. (2022). Digital Light Processing (DLP) of Yttria-Stabilized-Zirconia (YSZ). Master’s Thesis.

[63] Manafi S.A., Yazdani B., Rahimiopour M.R., Sadrnezhaad S.K., Amin M.H., Razavi M. (2008). Synthesis of nano-hydroxyapatite under a sonochemical/hydrothermal condition. Biomed. Mater..

[64] Lee D.S.H., Pai Y., Chang S. (2013). Effect of thermal treatment of the hydroxyapatite powders on the micropore and microstructure of porous biphasic calcium phosphate composite granules. J. Biomater. Nanobiotechnol..

[65] Bohner M., Le Gars Santoni B., Döbelin N. (2020). β-tricalcium phosphate for bone substitution: Synthesis and properties. Acta Biomater..

[66] Yashima M., Sakai A., Kamiyama T., Hoshikawa A. (2003). Crystal structure analysis of β-tricalcium phosphate Ca_3_(PO_4_)_2_ by neutron powder diffraction. J. Solid State Chem..

[67] Lin H.K., Pan Y.H., Salamanca E., Lin Y.T., Chang W.J. (2019). Prevention of bone resorption by ha/β-TCP + collagen composite after tooth extraction: A case series. Int. J. Environ. Res. Public Health.

[68] Phuong N.T., Nam N.H., Hong C.T., Dac D.V.Q., Thu L.P., Hai D.T., Osial M., Giersig M., Thanh D.T.M. (2023). Apatite Ore-based Nanostructures: Novel and Eco-friendly Sorbent for Efficient Removal of Wastewater Containing Pb^2+^ and Fe^3+^. Water Air Soil Pollut..

[69] Lazić S., Zec S., Miljević N., Milonjić S. (2001). The effect of temperature on the properties of hydroxyapatite precipitated from calcium hydroxide and phosphoric acid. Thermochim. Acta.

[70] Jagadale P.N., Jagtap P.P., Joshi M.G., Bamane S.R. (2016). A prototype synthesis and characterization of hydroxyapatite bioceramics nanocrystallites. Adv. Mater. Lett..

[71] Safarzadeh M., Ramesh S., Tan C.Y., Chandran H., Ching Y.C., Noor A.F.M., Krishnasamy S., Teng W.D. (2020). Sintering behaviour of carbonated hydroxyapatite prepared at different carbonate and phosphate ratios. Bol. Soc. Esp. Ceram. Vidr..

[72] Román F., Colomer P., Calventus Y., Hutchinson J.M. (2018). Study of hyperbranched poly(ethyleneimine) polymers of different molecular weight and their interaction with epoxy resin. Materials.

[73] Grenda K., Idström A., Evenäs L., Persson M., Holmberg K., Bordes R. (2022). An analytical approach to elucidate the architecture of polyethyleneimines. J. Appl. Polym. Sci..

[74] Feng C.F., Khor K.A., Gu Y.W., Cheang P. (2001). Analysis of phase changes in plasma-sprayed Ti-6Al-4V/hydroxyapatite composite coatings by DSC. Mater. Lett..

[75] Payne M.E., Lou Y., Zhang X., Sahiner N., Sandoval N.R., Shantz D.F., Grayson S.M. (2020). Comparison of cross-linked branched and linear poly(ethylene imine) microgel microstructures and their impact in antimicrobial behavior, copper chelation, and carbon dioxide capture. ACS Appl. Polym. Mater..

[76] Chang M.C., Tanaka J. (2002). FT-IR study for hydroxyapatite/collagen nanocomposite cross-linked by glutaraldehyde. Biomaterials.

[77] Abidi S.S.A., Murtaza Q. (2014). Synthesis and characterization of nano-hydroxyapatite powder using wet chemical precipitation reaction. J. Mater. Sci. Technol..

[78] Prekajski M., Mirković M., Todorović B., Matković A., Marinović-Cincović M., Luković J., Matović B. (2016). Ouzo effect—New simple nanoemulsion method for synthesis of strontium hydroxyapatite nanospheres. J. Eur. Ceram. Soc..

[79] Jiang B., Ke S., Yang B., Chen J., Li W., Fang M., Huang Z., Sun J., Min X., Hu X. (2023). Interfacial characteristics of bioglass diffusion zone between scaffold-like hydroxyapatite/wollastonite coating and dense zirconia substrate. Ceram. Int..

