# Supplementary material for: Hydroxyapatite-Based Coatings on Silicon Wafers and Printed Zirconia
Source: J Funct Biomater. 2023 Dec 27;15(1):11. doi: 10.3390/jfb15010011 (PMC10817446; doi:10.3390/jfb15010011)
Supplement: Supplementary file 1 [file jfb-15-00011-s001.zip › jfb-2705013-supplementary.pdf]

Hydroxyapatite-Based Coatings on Silicon Wafers and Printed Zirconia

Antoine Chauvin [1], Marie-Rose Garda [1], Nathan Snyder [2], Bai Cui [2], Nicolas Delpouve [1,*] and Li Tan [2]

[1] Groupe de Physique des Matériaux UMR 6634, CNRS, Université de Rouen Normandie, INSA Rouen Normandie, F-76000 Rouen, France; marie-rose.garda@univ-rouen.fr (M.-R.G.)
[2] Department of Mechanical and Materials Engineering, University of Nebraska, Lincoln, NE 68588, USA; bcui@unl.edu (B.C.); ltan4@unl.edu (L.T.)

* Correspondence: nicolas.delpouve1@univ-rouen.fr; Tel.: +33-(0)232955165

Supporting Information:

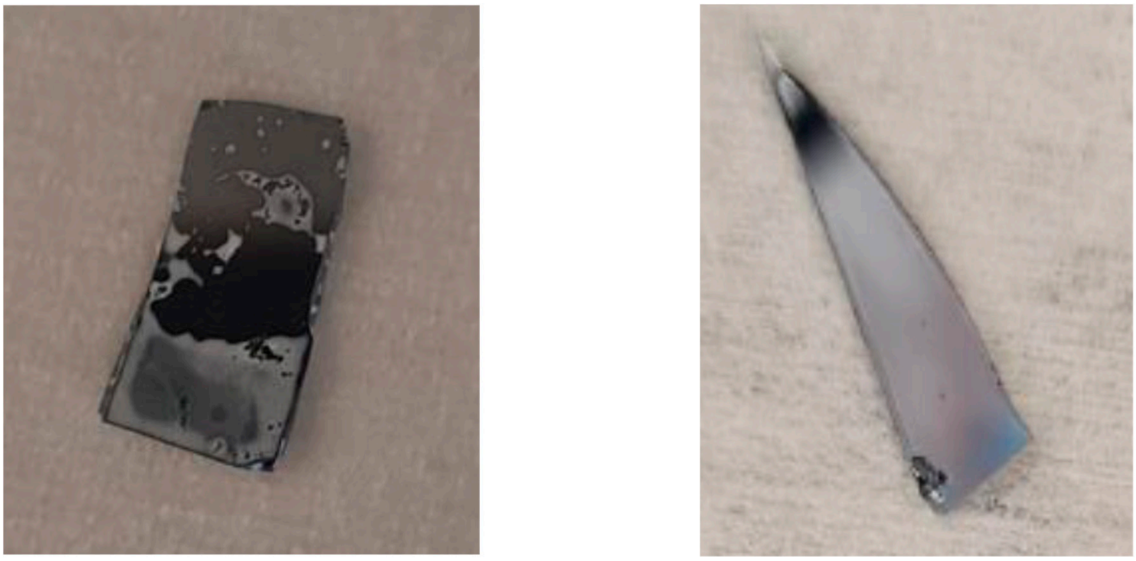

(a) (b)

Figure S1: Surface state after the removal of a piece of tape stuck on the coating. In this case, the HAp coating (a) did not bear the tearing solicitation, while the PEI/HAp coating (b) showed better adhesion.
